# Implementing a WASH Package Within Prevention of Vertical Transmission of HIV Programming in Lilongwe, Malawi: A Program Evaluation

**DOI:** 10.5334/aogh.5322

**Published:** 2026-07-14

**Authors:** Hillary M. Topazian, Skyler Noble, Innocent Mofolo, Gertrude Mwale, Twambilie Phanga, Irving F. Hoffman, Michael E. Herce

**Affiliations:** 1 University of Utah, Salt Lake City, United States; 2 University of North Carolina, Chapel Hill, United States; 3 UNC Project, Lilongwe, Malawi

**Keywords:** WASH, water, sanitation, hygiene, prevention of vertical transmission of HIV, program implementation

## Abstract

*Background:* Access to safe drinking water is a considerable problem in Malawi. Contaminated water plays a major role in diarrheal disease, particularly for people living with HIV and their families. Water, sanitation, and hygiene (WASH) interventions can reduce the risk of diarrhea among children by up to 50%. However, few published reports describe WASH integration in routine HIV care programming in Africa.

*Objective:* To evaluate a WASH program integrated within the prevention of vertical transmission of HIV (PVTH) care in Lilongwe, Malawi.

*Methods:* The University of North Carolina’s Project Malawi, the Proctor & Gamble Company (P&G), and the Malawi Ministry of Health implemented a safe drinking water program from 2008 to 2023, integrated within the national PVTH program in Lilongwe, Malawi. We provided a point-of-use WASH package to pregnant and breastfeeding women (PBFW) with HIV and their families that included soap, sieves, closed-water containers, and P&G water purification packets. We examine program impact by describing trends in childhood diarrhea and retention in care at 33 program facilities and 29 non-program facilities from 2018 to 2022.

*Findings:* Over the program’s final 5 years, an estimated 2 million water purification packets were distributed annually. From 2018 to 2022 in program facilities, non-bloody diarrhea rates among children under 5 years remained constant, while bloody-diarrhea rates fell at both program and non-program facilities. A small association was found between facility participation in the program and retention in care among PBFW living with HIV (incidence rate ratio: 1.05, 95% CI: 0.99–1.12), but of borderline statistical significance.

*Conclusions:* In an environment where access to clean water is not assured, we demonstrated the feasibility of delivering a WASH package in HIV care settings over 15 consecutive years. The collaboration between government, academic, and industry partners provides an example for how to integrate WASH within PVTH programming.

## Introduction

Access to safe drinking water is a fundamental human right [1] and a crucial component of public health. In sub-Saharan Africa (SSA), 408 million people lack at least basic drinking water services, and only 31% of people have safely managed drinking water—that is water that is accessible, available when needed, and free from fecal and chemical contamination—compared to 73% of the global population [2]. The United Nations has set ambitious global development targets for clean water and sanitation, including Sustainable Development Goal 6, which aims to achieve “universal and equitable access to safe and affordable drinking water” by 2030 [3]. However, achieving this goal will require addressing complex socioeconomic, environmental, and water infrastructure-related factors, particularly in low- and middle-income countries, where clean water infrastructure and access are limited.

Contaminated water plays a leading role in transmitting diarrheal diseases, including cholera, dysentery, hepatitis A, polio, and typhoid. Each year approximately 842,000 people die globally from diarrheal diseases due to unsafe drinking water and sanitation [4]. It is thought that 143,300 deaths among children under 5 years in SSA were attributed to unsafe water in 2017 [5]. Diarrhea is the third leading cause of mortality in children under 5 years in low- and middle-income countries and is responsible for half a million child deaths each year [6]. The burden is even more pronounced in children living with HIV, as diarrheal disease occurs more frequently and with more severe outcomes in this population [7]. Cross-sectional survey data across SSA have shown that 18% of children under 5 years have had diarrhea in the previous two weeks, with urbanized areas, poorer populations, and lack of improved water and toilet facilities all contributing to higher risk of diarrhea [8].

In Malawi, a resource-constrained country in SSA, diarrhea is common among children. The 2024 Malawi Demographic and Health Survey found that 22% of children under age 5 had diarrhea in the 2 weeks before the survey and that 20% of these children received no treatment [9]. Eighty-eight percent of Malawian households get their drinking water from an improved source such as piped water, public taps, boreholes, and protected water sources, among others. The primary sources of drinking water for urban households in Malawi are piped water to a dwelling or yard (43%) or public taps or standpipes (22%). Rural Malawian households have less access to improved water sources and the majority use tube wells or boreholes (65%) or unimproved water sources (9%) [9]. As a result, water must often be collected manually, frequently from open sources, and 57% of households do not treat their drinking water. Twenty-one percent of rural households travel more than 30 minutes to obtain drinking water [9], and the burden of water collection falls disproportionately on women [10], impacting gender equality, household dynamics, and economic productivity.

Recent reductions in child mortality from diarrheal diseases have occurred in part because of improved water, sanitation, and hygiene (WASH) interventions. Evidence suggests that WASH interventions such as treating water through filtration, solar, and chlorination, and providing an improved drinking water supply, can reduce the risk of diarrhea in children by 50% [11], and reduce mortality by 45% in children under 5 years [12]. Similarly, WASH programs have been shown to reduce morbidity among adults and children living with HIV, counteracting the effects of contaminated water from which diarrhea can result in increased viral load, decreased CD4 counts, and clinical decline [13].

Malawi was the first country to implement Option B+ in its national prevention of mother-to-child transmission program in 2011 (now referred to as prevention of vertical transmission of HIV, or “PVTH”), offering life-long antiretroviral therapy (ART) to all pregnant and breastfeeding women (PBFW) with HIV regardless of CD4 count [14]. A national survey in 2020–2021 reported that 5.7% of women giving birth within the previous year tested HIV positive, leading to a large population which qualifies for PVTH [15]. Research has shown that incentives such as cash transfers can improve retention in PVTH programs [16], but little information exists on providing integrated WASH services as an incentive for retention.

The University of North Carolina’s (UNC) Project Malawi, in partnership with the Proctor & Gamble Company (P&G) and the Lilongwe District Health Office (DHO) of the Ministry of Health (MOH), launched a clinic-based, safe drinking water program in 2008 (“the Safe Water Program” or the “program”) with the goal of providing clean drinking water to communities in Lilongwe district, Malawi. This program was integrated within the national PVTH program through HIV technical assistance projects funded by the United States Agency for International Development and the United Nations Children’s Fund (UNICEF). The Safe Water Program was designed to alleviate the burden of diarrheal diseases among PBFW living with HIV, their infants and children, and their families, while also incentivizing engagement with the Malawi PVTH program. An early program evaluation from October 2009 to March 2010 showed high uptake of WASH commodities (99.4%), high retention in HIV care for PBFW, and diarrheal rates lower than those reported nationally, with only 17.7% of infants having at least one episode of diarrhea over the previous three months [17].

In this paper, we describe the overall reach and performance of the program. We also conducted a follow-on serial cross-sectional study at program and non-program facilities in Lilongwe District during the last 5 full years of the program, from 2018 to 2022, to evaluate the program’s effects on childhood diarrheal disease incidence, retention in care for PBFW living with HIV, and program coverage among all women enrolled in PVTH. Our overall aim is to provide programmatic evidence for integrating WASH interventions into routine PVTH programming to improve the health of PBFW living with HIV and their families.

## Methods

### Program design and population

The Safe Water Program initially supported 2 large public health centers offering PVTH services in 2008 and then expanded its reach to serve 33 hospitals and health centers throughout Lilongwe District by 2013. Hospitals in Lilongwe are generally much larger than health centers and have an inpatient facility, whereas health centers primarily serve outpatients, except for inpatient maternity wards. The program was integrated within the national PVTH program through HIV technical assistance projects, which supported the Malawi MOH to eliminate vertical transmission through staff training, promoting male partner involvement, encouraging women’s community-based psychosocial support groups, and strengthening laboratory infant HIV testing systems [18]. The initial aims of the Safe Water Program were to reduce diarrheal disease among PBFW living with HIV and their infants and children, and to encourage HIV care retention by providing a tangible WASH package at each monthly visit. Program beneficiaries included infants exposed to HIV who were up to 2 years of age, PBFW living with HIV, and other family members. PBFW were screened for program eligibility during post-natal and ART visits and identified by PVTH program staff. The facilities that were chosen to participate in the Safe Water Program were selected purposively based on facility assessment, including availability of on-site PVTH services, on-site secure storage for commodities, prioritizing more rural and peri-urban areas, and catchment populations where most people used untreated water.

### Interventions

**Figure 1:**
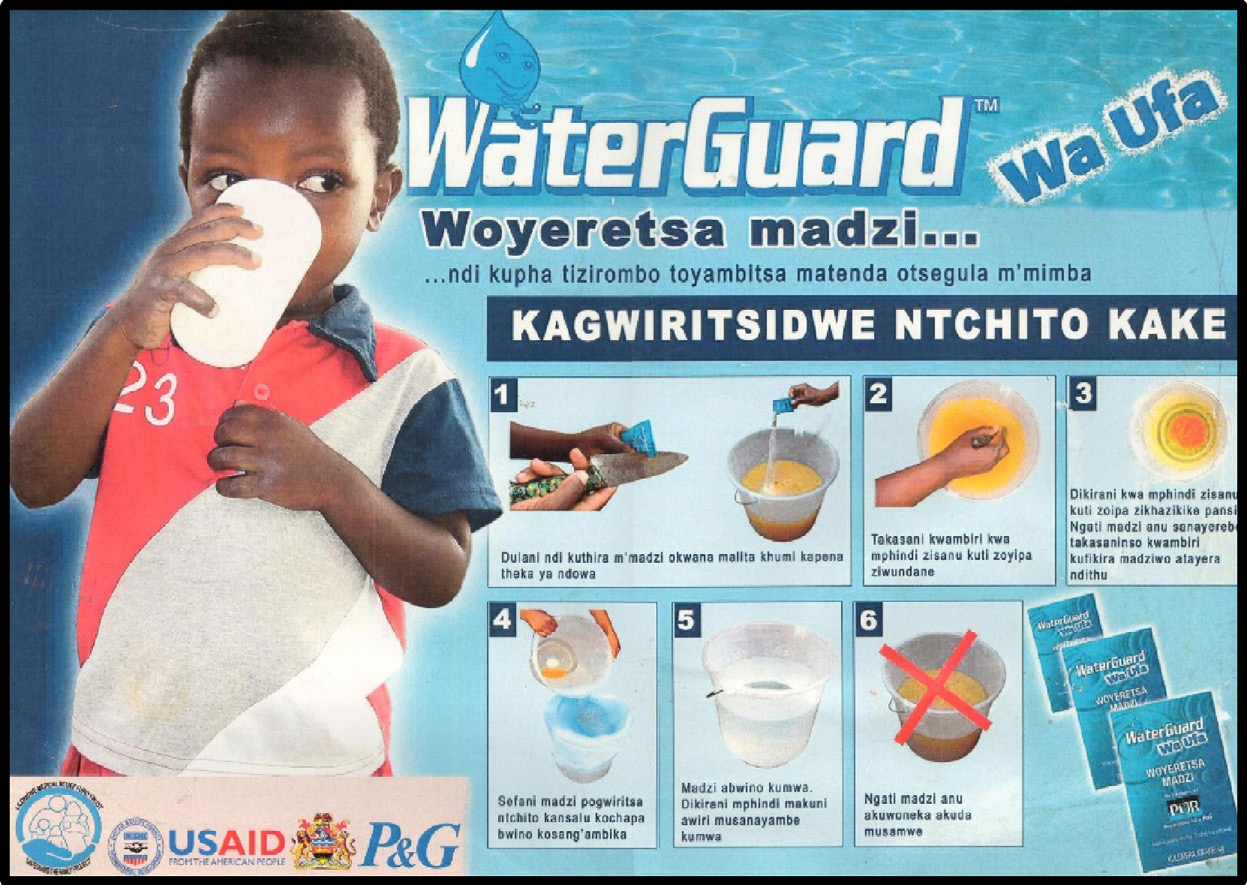
An educational poster used by program facilities. Text is written in Chichewa and describes how to use P&G water purification packets, buckets, and sieves to purify water.

Safe Water Program beneficiaries received a WASH package that included health education and materials for water purification, including soap, water storage buckets, and sieves, and clean drinking water supplies in the form of P&G water purification packets (Figure 1). Participants were provided with the bucket, cloth, soap, and the water purification packets upon enrollment; then, 120 water purification packets were received monthly with additional cloth and soap distributed as needed. Only one bucket per family was provided. The P&G water purification packet is a 4-g sachet containing a powdered combination of ferric sulfate coagulant and calcium hypochlorite disinfectant that can purify 10 liters of water [19]. The P&G water purification packet has been classified by the World Health Organization as providing comprehensive protection against bacteria, viruses, and protozoa.

Women attending PVTH and post-natal clinics at program facilities were offered the WASH package. HIV-exposed infants, young children, and their mothers who were identified from under-5 clinics, outpatient departments, and HIV treatment clinics at the facilities were encouraged to attend the PVTH clinic and enroll in the Safe Water Program. Distribution, sensitization, and training of participants regarding the use of clean drinking water supplies were provided daily by government nurses and counselors based at each clinic. Women continued to be enrolled in the program through the breastfeeding period, usually about 2 years or at the discretion of the clinic staff. Distribution of the WASH package was fully aligned with beneficiaries’ schedule of routine PVTH visits to promote the uptake of the intervention and retention in HIV care, under-5 pediatric care, and other primary care services.

### Data collection

From 2018 to 2022, MOH staff at each program facility recorded aggregated count data on PVTH program registrations, Safe Water Program enrollments, water purification packets distributed, and ART program status (i.e., retained in care, transferred out, died, and otherwise lost to follow-up). UNC Project staff reviewed the documented program registries and visited facilities to access and cross-check physical records during monthly supportive supervision visits.

**Figure 2:**
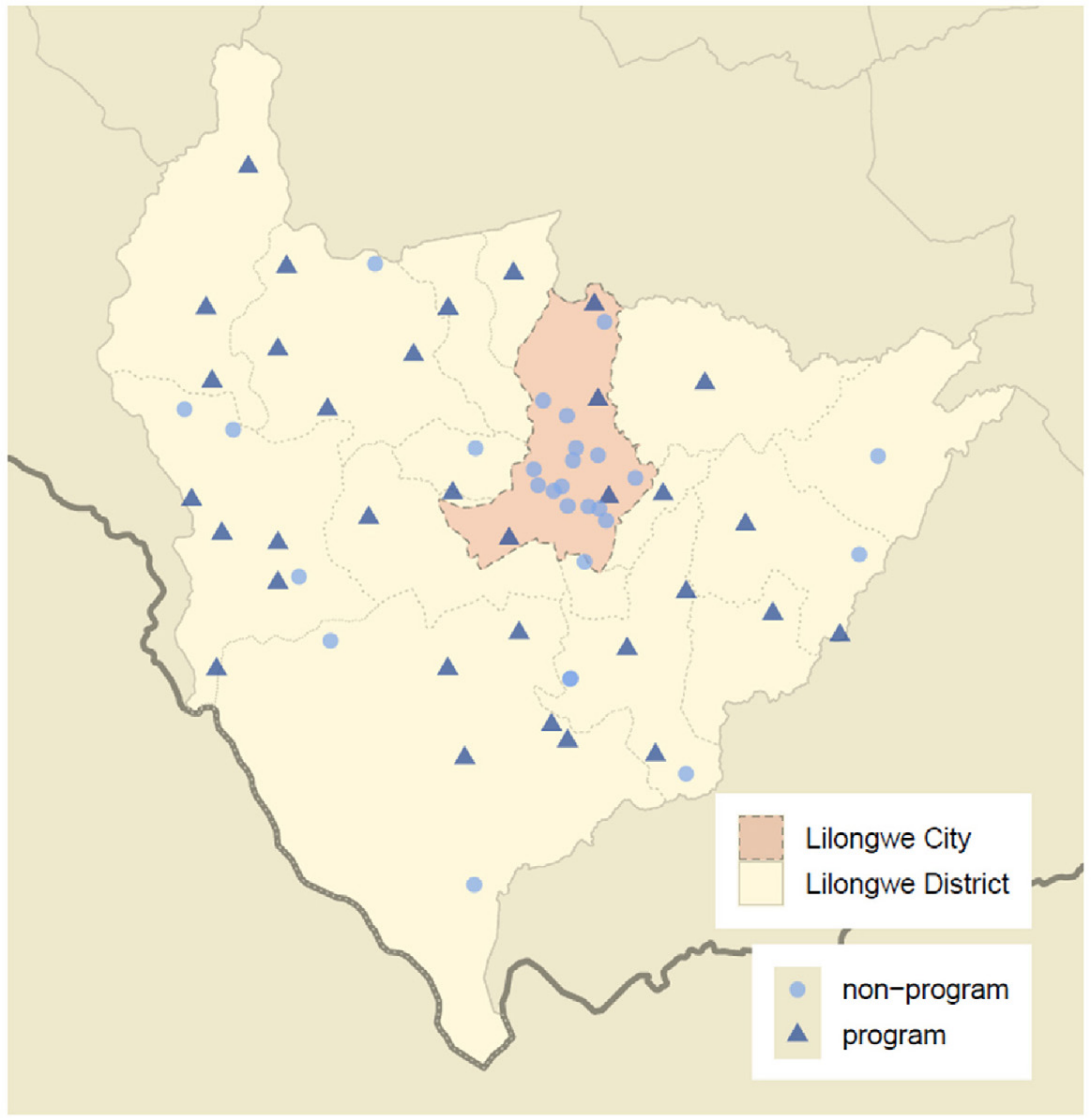
Geographical depiction of Safe Water Program facilities in Lilongwe, Malawi. Facility locations are depicted as points; light blue circles indicate non-program facilities (*n* = 29); dark blue triangles indicate program facilities (*n* = 33). The dark gray line indicates the Malawi-Mozambique border.

DHIS2 is the national data management system for monitoring diarrheal diseases and other public health indicators at facility, district, provincial, and national levels. Data are documented prospectively by health facilities and then aggregated monthly as counts of cases and deaths. There is no tracking of individuals in these data, meaning that one person could contribute multiple times to case counts over a reporting period. The Lilongwe DHO of the MOH provided routine data for diarrheal cases, dysentery cases, and deaths for children under 5 years of age through the national DHIS2 system. The Lilongwe DHO had data available for 25 of the 33 program facilities included in this analysis and all 29 non-program facilities in Lilongwe District that did not participate in the Safe Water Program (Figure 2). Eight Safe Water Program facilities did not have enough data collected over the study period to evaluate and were not included in the analysis. The Lilongwe DHO and facility data offices also provided estimates of the under-5 catchment populations for program and non-program facilities. The Lilongwe DHO provided information to classify each facility’s water and electricity access, health system level (health center, hospital), ownership (government, private), urbanicity (urban, rural), and geographic coordinates. Each program facility provided information on the number of clinical staff (doctors, clinicians, medical assistants, nurses, and community midwives) employed in the year 2024; unfortunately, historical data for these indicators were not available by year. The cross-sectional impact analysis was based on data from 2018 to 2022 only, as this was the period with the most complete and well-specified MOH and program data.

The Monitoring and Evaluation Unit in the Directorate of HIV, STI, and Viral Hepatitis at the Malawi MOH provided data on PVTH enrollments from facilities across Malawi. Variables included counts of total facility PVTH registrations and quarterly PVTH program status, including the number of women who were alive and retained on ART, women who were lost to follow-up (LTFU), women who were still in care, but who had stopped ART, and women who had transferred to another facility. All data were reported quarterly, and counts were averaged to produce annual estimates; there was no tracking of individuals.

### Outcomes

The primary outcome of this sequential cross-sectional analysis was the annual incidence of non-bloody and bloody diarrheal disease among children under 5 years from 2018 to 2022, defined as the number of cases of diarrhea in children under 5 years reporting to each facility each year divided by the under-5 population count of the facility’s catchment area.

A secondary outcome of analysis was retention in the PVTH program, defined as the quarterly proportion of PBFW living with HIV and registered in the PVTH program who remained active ART users (excluding those who had transferred out). Another secondary outcome of interest was the reach of the Safe Water Program, defined for each program site as the total number of beneficiaries divided by the number of PBFW living with HIV and enrolled in PVTH at each program site each year.

### Statistical analysis

We summarized the changes in diarrheal incidence among the under-5 population at program and non-program facilities between 2018 and 2022. Descriptive statistics were calculated for all facilities based on facility type, urbanicity, and facility ownership variables, with counts reported for categorial variables and means and standard errors for continuous variables. Descriptive statistics for program facilities also included water and electricity access, catchment population of children under 5 years, and facility staff variables. Statistical significance was measured with a threshold of *P* ≤ 0.05.

### Impact evaluation

Multivariate associations between facility participation in the Safe Water Program and diarrheal and dysentery incidence among children were conducted using zero-inflated negative binomial regression models due to overdispersion of diarrhea cases and presence of zeros. Models accounted for facility-level clustering using random intercepts and fixed effects for program group and year, while the adjusted models additionally controlled for the following facility characteristics: urban/rural location and facility type. An offset term was included for the log of the estimated catchment population of children under 5 years at each facility to account for variation in facility catchment size. The models assumed constant zero-inflation probability across observations. A zero-inflated negative binomial regression model was also run to look at the bivariate association between facility participation in the Safe Water Program and facility-level ART retention. An offset term was included for the total number of clients at each facility.

All statistical analyses were performed using R V.4.4.2 (R Foundation for Statistical Computing, Vienna, Austria). Mapping was completed using the sf (v1.0-15; Pebesma, 2023) package and zero-inflated negative binomial model diagnostics were performed using the DHARMa (v0.4.7, Hartig, 2024) package.

### Ethical considerations

This impact analysis was nested within a larger program evaluation that was provided ethical exemption by the Malawi National Health Sciences Research Committee (protocol 17/01/1714) and the University of North Carolina at Chapel Hill (study 17-0030) for this review of routine, aggregated and de-identified program data.

## Results

**Figure 3:**
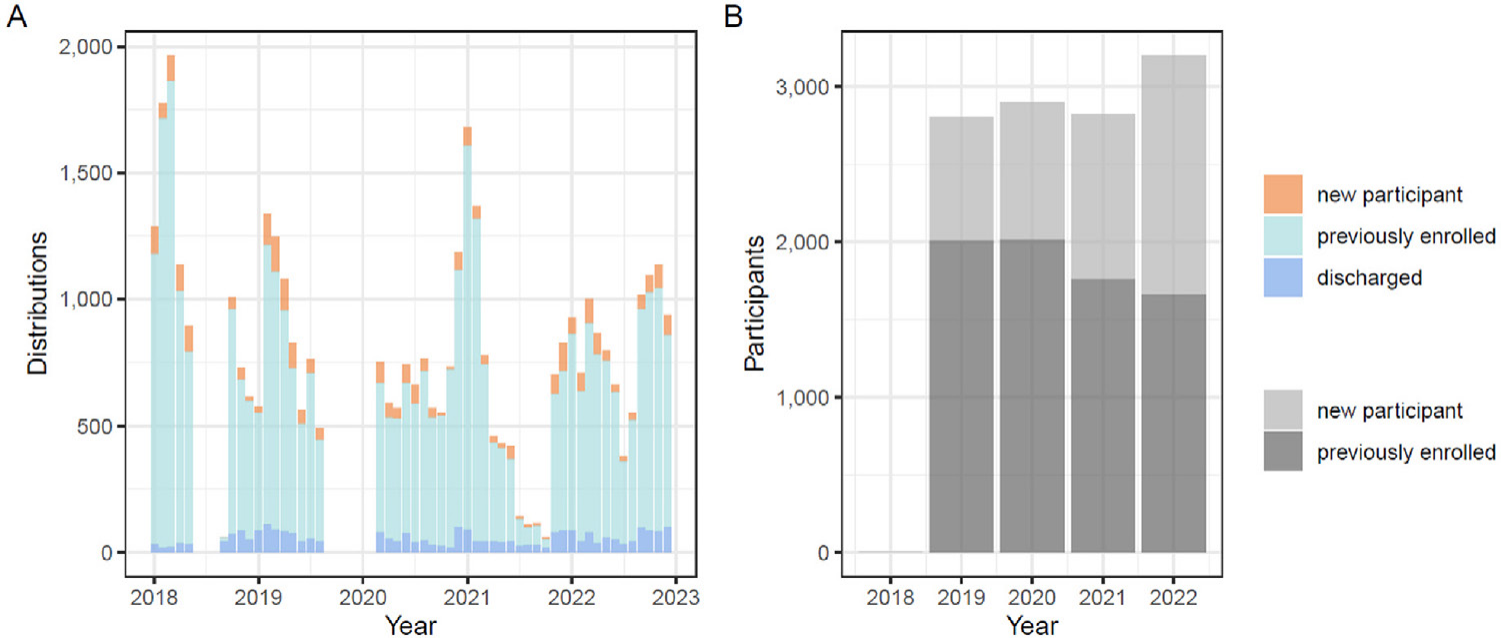
Program distribution and enrollment characteristics from January 2018 to December 2022. Panel **(A)** shows monthly kit distribution counts at program facilities, stratified by participant status. Months where no data were reported are due to commodity stock outs or fuel shortages impairing distribution. Non-program facilities that distributed materials in 2018 in response to cholera and flooding are not represented on the plot. Panel **(B)** shows counts of all participants who participated in the program; data were unavailable for 2018.

The Safe Water Program distributed 43,910 kits and over 10 million water purification sachets from January 2018 to December 2022 and reached an average of 2,900 women per year (Figure 3). Given an average household size of 4.5 people [9], we estimate that 13,200 people were reached by the program annually over this period. In addition to the kits distributed by program facilities, three non-program facilities distributed 3,263 kits (25.6%) in 2018 in response to widespread flooding and cholera outbreaks. We estimate that the under-5 catchment populations for the 25 Safe Water Program facilities with data available included over 481,000 infants and children. We further estimate that if each woman participating in the program had 4.4 children [9], the national average in Malawi, the program would have reached over 12,700 children per year, which represents less than 3% of our facility under-5 catchment population.

A total of 33 program facilities and 29 non-program facilities were included in the impact evaluation analysis from 2018 to 2022 (Table 1). The majority of facilities in both groups were classified as health centers rather than hospitals, including 24 (72.7%) in the program group and 24 (82.8%) in the non-program group. Groups had a significant difference in urbanicity classification, with 28 (84.8%) program facilities and 14 (48.3%) non-program facilities classified as rural, respectively. All the non-program facilities were government run, while 25 (75.8%) program facilities were public. All program facilities with data available (*n* = 25, 75.8%) had water and electricity access. Program and non-program facility groups had a wide range of catchment population values, with program facilities having a mean of 16,038 (standard error [SE]: 20,913) children under 5 years, and non-program facilities having a mean of 10,116 (SE: 11,985). Program facilities had a mean of 0.7 (SE: 1.8) doctors, 3.1 (SE: 4.8) clinical officers, 2.8 (SE: 1.7), medical assistants, 14.6 (SE: 18.1) nurses, and 3.9 (SE: 10.3) community midwives.

**Table 1:** Characteristics of Safe Water Program facilities and non-program facilities.

	VARIABLE	PROGRAM FACILITIES		NON-PROGRAM FACILITIES		*P*-VALUE
		*N*	*%*	*N*	*%*	
**Total number**		33		29		
Facility type*	Health center	24	72.7	24	82.8	0.2
	Hospital	6	18.2	5	17.2	
**Urbanicity**	Rural	28	84.8	14	48.3	<0.001
	Urban	2	6.1	15	51.7	
**Ownership**	Government	25	75.8	29	100	0.02
	Private	5	15.2	0	0	
		***N***	***%***	***N***	***%***	
**Water access**		25	75.8	–	–	
**Electricity access**		25	75.8	–	–	
		***MEAN***	***SE***	***MEAN***	***SE***	
**Catchment population, children under 5 years^†^**		15,936	20,932	10,029	11,825	0.2
**Facility staff**	Doctors	0.7	1.8	–	–	
	Clinical officers	3.1	4.8	–	–	
	Medical assistants	2.8	1.7	–	–	
	Nurses	14.6	18.1	–	–	
	Community midwives	3.9	10.3	–	–	

*Note:*missing data - three program facilities are missing data on facility type, urbanicity, ownership, and catchment population. Eight program facilities are missing data on water access, electricity access, and facility staff.

*Hospitals are larger than health centers and have inpatient facilities. Health centers are primarily outpatient facilities although most have inpatient maternity services.

^†^The catchment population of children under 5 years for each facility was averaged over the years 2018–2022 if facilities reported populations yearly.

**Figure 4:**
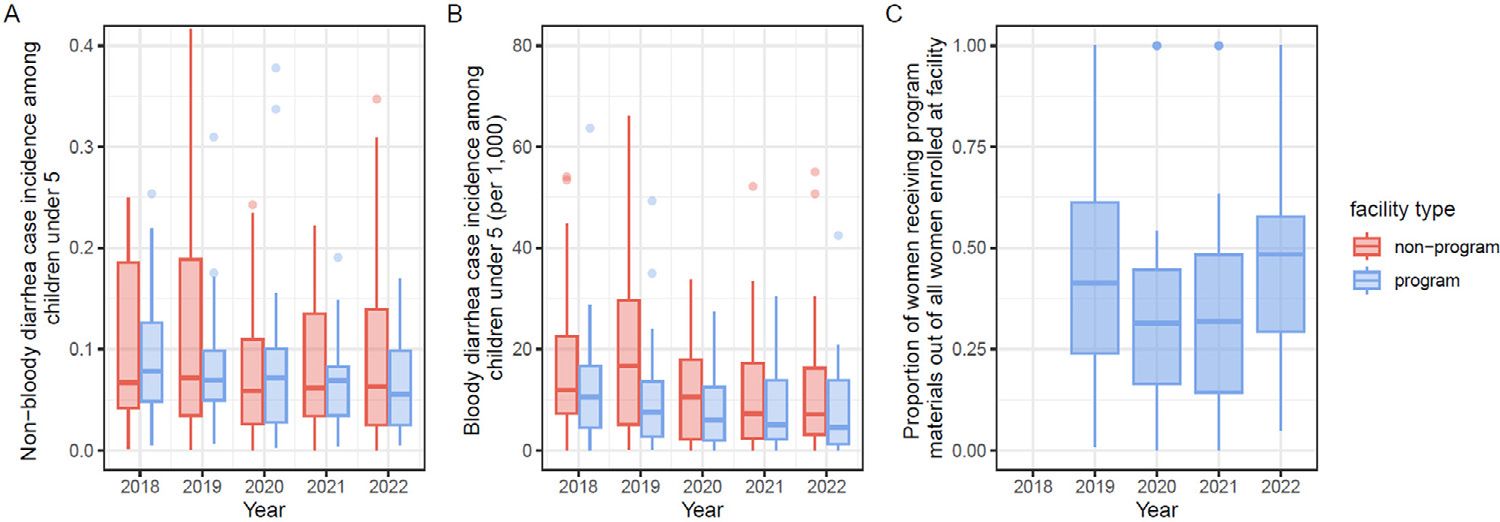
Annual case incidence of **(A)** non-bloody diarrhea and **(B)** bloody diarrhea among children under 5 years from 2018 to 2022, stratified by program and other non-program facilities (*n* = 300). Panel **(C)** represents the annual coverage of the Safe Water Program, the proportion of women benefitting from the program out of all women enrolled in PVTH at program facilities; data were unavailable for 2018. Ten observations fall outside plot area A with non-bloody diarrhea incidence in children > 0.4 and 3 observations fall outside plot area B with bloody diarrhea incidence in children > 80 per 1,000. Thirty-one observations (facilities in a given year) are missing non-bloody diarrhea data, 32 observations are missing bloody diarrhea data, and 50 observations are missing coverage data.

Annual case incidence rates of non-bloody diarrhea in children under 5 years fluctuated over time but remained relatively constant. Median values in the program group ranged from 0.055 to 0.078 cases per person per year and in the non-program group from 0.059 to 0.071 cases per person per year (Figure 4A). Annual case incidence rates of bloody diarrhea in children under 5 years declined in both groups (Figure 4B). In program facilities, rates fell from a median of 10.6 cases per 1,000 people per year in 2018 to 4.6 in 2022 and from 11.9 in 2018 to 7.1 in 2022 in the non-program group. Program coverage in the catchment population of interest, defined as the proportion of women benefitting from the program out of all women enrolled in PVTH at program facilities, ranged from 31% in 2020 to 48% in 2022 (Figure 4C).

**Figure 5:**
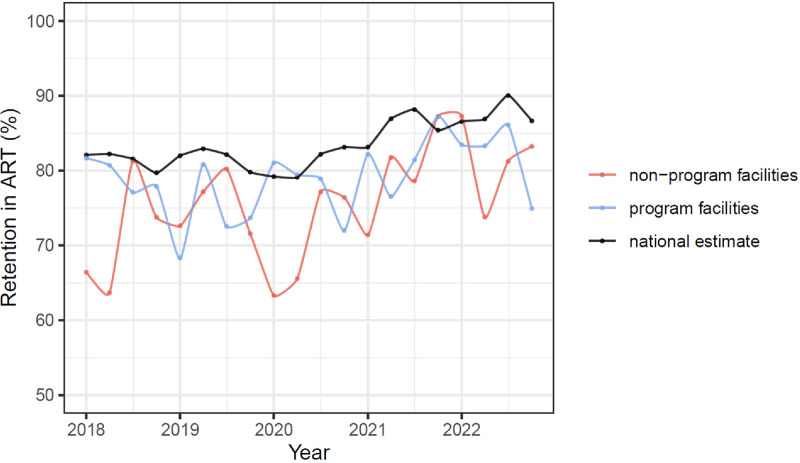
Antiretroviral therapy (ART) retention plotted quarterly from 2018 to 2022. Results are stratified by program facilities (*n* = 33) and non-program facilities (*n* = 29). Lines represent the mean quarterly ART retention values for program facilities, non-program facilities, and a national estimate (*n* = 874).

From 2018 through the beginning of 2022, ART retention for PBFW living with HIV was stable nationally. ART retention fluctuated between 68.3% and 87.2% at program facilities and 63.3% and 87.2% at non-program facilities (Figure 5). Twenty-nine percent of participants who were LTFU self-reported transferring to another clinic for care.

We found no statistically significant association between facility participation in the Safe Water Program and any of the three outcomes assessed. For non-bloody diarrhea incidence, the incidence rate ratio was 0.99 (95% CI: 0.54–1.83, *P* = 1.0) in the unadjusted model and 1.20 (95% CI: 0.62–2.34, *P* = 0.6) after adjusting for urbanicity and facility type (Table 2). For bloody diarrhea incidence, the incidence ratio was 0.61 (95% CI: 0.32–1.16, *P* = 0.10) in the unadjusted model and 0.81 (95% CI: 0.41–1.63, *P* = 0.6) after adjusting for urbanicity and facility type. ART retention was higher at program facilities compared to non-program facilities, but this difference did not reach statistical significance after adjusting for urbanicity and facility type (IRR = 1.05, 95% CI: 0.99–1.12), *P* = 0.09).

**Table 2:** Bivariate and multivariate associations between facility participation in the Safe Water Program and diarrheal incidence among children under 5 years, 2018–2022, and antiretroviral therapy (ART) retention from 2018 to 2022.

OUTCOME	MODEL	ESTIMATE (INCIDENCE RATE RATIO)	95% CONFIDENCE INTERVAL	*P*-VALUE
Non-bloody diarrhea incidence	Unadjusted	0.99	0.54–1.83	1.0
	Adjusted*	1.20	0.62–2.34	0.6
Bloody diarrhea incidence	Unadjusted	0.61	0.32–1.16	0.1
	Adjusted*	0.81	0.41–1.63	0.6
ART retention	Unadjusted	1.06	1.01–1.10	0.008
	Adjusted*	1.05	0.99–1.12	0.09

*Adjusted for urbanicity and facility type.

## Discussion

We implemented a Safe Water Program to provide a WASH package integrated within a routine PVTH program to reduce the burden of diarrheal disease among PBFW living with HIV and their infants and children exposed to HIV in Lilongwe, Malawi. We observed high annual enrollments and high ART retention. While we did not observe a statistically significant association at the health facility level between program receipt and declines in childhood diarrheal disease incidence or improved maternal ART retention, our descriptive data suggest that the program did not disrupt overall secular trends toward declining childhood diarrheal disease incidence and may have improved maternal ART retention to a small degree that we did not have power to detect in this study. Taken together, these data suggest the feasibility of an approach that offers basic WASH services in routine HIV care and maternal health settings in Malawi.

Our analysis of the Safe Water Program demonstrated a high distribution in Lilongwe, Malawi, with over 43,910 safe drinking water kits distributed by 33 program facilities during the last 5 years of the program’s 15-year history. An average of 2,900 women were enrolled per year over the study period.

From 2018 to 2022, annual non-bloody diarrhea case incidence among children fluctuated over time, while bloody diarrhea case incidence decreased, in both program and non-program groups. An early program evaluation from October 2009 to March 2009 showed high uptake of WASH products (99.4%), moderate retention in care for women (75.3%) at 3 months follow-up, and lower infant diarrheal rates (17.7%) than those reported nationally [17]. Numerous randomized controlled trials across Africa, Asia, and the Americas, have also shown that water disinfection at point-of-use reduces individual-level diarrhea risk among young children, with a pooled relative risk of 0.69 (95% CI: 0.60–0.79) across 44 studies published before 2016 [20]. A more recent systematic review of studies published between 2016 and 2021 showed that water treatment at point-of-use reduced childhood diarrhea risk by 50% using filtration and 44% using chlorination compared to an unimproved water source [11]. These relationships also hold for WASH interventions among people living with HIV [13].

However, our findings showed no association between facility-level program enrollment and population-level non-bloody diarrhea and bloody diarrhea incidence among children over the study period, likely due to a complex relationship between program reach and disease incidence. One hypothesis for why we did not observe a similar risk reduction in our study is the relatively low population penetration of our integrated WASH intervention into the Lilongwe metro area (~3%), where the population increased by more than 50% between 2013 and 2023 [21]. Publication of two large factorial randomized controlled trials in Kenya [22] and Zimbabwe [23] also showed no population-level effect on diarrhea after distributing individualized WASH interventions, thought to be because the intervention households or clusters only represented a small fraction of the community contributing to population-level diarrhea incidence [24]. A similar trial conducted in Bangladesh [25] showed a large population-level diarrhea relative risk reduction; however, this effect only held for sanitation, handwashing, nutrition, or combination treatments, not for children receiving only water treatments as in our program. The discrepancy in findings between countries was thought to be due to unique pathogens in each of the settings which may be differentially affected by WASH interventions, local etiologies of diarrhea, and differing environmental pathogen transmission pathways [24]. Reducing population-level diarrheal burden in this urbanizing setting will require interventions that can scale alongside population growth and district-wide WASH programming that can reach the majority of households, not only those attending HIV care facilities.

Facility-level Safe Water Program participation was associated with a numerically higher but statistically non-significant care retention in our study population, with an adjusted incidence rate ratio of 1.05 (95: CI: 0.99–1.12; *P* = 0.09). Loss to follow-up in PVTH programs in SSA represents a major barrier to ending pediatric HIV globally. Studies from the 1990s and early 2000s estimated that PVTH loss to follow-up rates were between 19% and 89.4%, with rates increasing as women move from antenatal care to delivery to postpartum services [26]. Reasons cited for loss to follow-up included fear of HIV testing, stigma and discrimination, home deliveries rather than health facility births, and staff and supply shortages. Past research from Lilongwe, Malawi, has shown loss to follow-up rates of 85% at 3 months and 79% at 12 months, associated with younger age, unemployment, and ART initiation while pregnant [27]. More recent data suggest that nearly 30% of PBFW with HIV in Malawi experienced LTFU within 2 years after a new HIV diagnosis [28]. Twenty-nine percent of participants in our study population who were LTFU reported self-transferring to another clinic.

The rigor of our analysis was limited primarily by the quality and availability of routine facility and program data. Routine registers maintained by facility staff were designed for service delivery and not for research, leading to missing data and a lack of data collected at some pre-2018 facilities. Program facilities were selected based on perceived need (low-quality water sources) of the catchment populations particularly in relatively rural and peri-urban areas, and the relative patient volumes in the PVTH program. Without historical data prior to program commencement, or random assignment of facilities to program versus non-program status, we were unable to fully capture the effects of our intervention. Several facilities had incomplete or nonexistent records even during the study period, anecdotally citing a lack of incentives, time, and trained staff as reasons for poor documentation. Safe Water Program recipients were not tracked individually to determine accurate annual participation and enrollment counts, which limits our understanding of the coverage of our intervention in the target population. We also measured uptake of the intervention through the number of beneficiaries enrolled in the program; however, enrollment or distribution of clean water supplies does not guarantee proper use of the intervention. We were unable to adjust for other factors in our analysis that may confound the relationship between program participation and health outcomes. These include heath care quality and introduction of other WASH-focused interventions targeting communities during the study period. Examples include regional emphases on WASH guidelines following flooding and after cholera outbreaks in 2020 and 2023. Local populations’ access to water, transportation to health facilities, stable electricity, and financial security, may have differentially affected program and other non-program facilities leading to residual confounding. The intervention studied here did not address aspects of WASH beyond clean water, such as increasing access to and use of improved latrines and handwashing. It is likely that WASH interventions must concurrently address the effects of both animal and human waste to reduce diarrhea incidence in this population.

Despite these limitations, the Safe Water Program was able to integrate a WASH intervention into the national PVTH program in Lilongwe, reaching thousands of PBFW living with HIV and their families. Although we were unable to track individual outcomes, the program anecdotally provided unmeasured benefits, including supporting the Malawi MOH with WASH activities and PVTH care, integrating WASH and HIV services, and strengthening HIV and maternal health services delivery platforms. The collaboration between UNC, the MOH, and P&G broadened the distribution of clean drinking water supplies during emergencies, following the model set-up by the program. To change population-level rates of diarrhea among children in Lilongwe, a wide-scale, government-led, comprehensive package of interventions is needed that can target a larger number of health facilities and surrounding community members to control local pathogens and interrupt entrenched diarrheal disease transmission routes.
